# Effect of RAAS blockers on adverse clinical outcomes in high CVD risk subjects with atrial fibrillation: A meta-analysis and systematic review of randomized controlled trials

**DOI:** 10.1097/MD.0000000000004059

**Published:** 2016-07-01

**Authors:** Sandip Chaugai, Lhamo Yanchang Sherpa, Amir A. Sepehry, Hisatomi Arima, Dao Wen Wang

**Affiliations:** a Department of Internal Medicine and Institute of Hypertension, Tongji Hospital, Tongji Medical College, Huazhong University of Science and Technology, Wuhan, China; b Department of Community Medicine, Section of Preventive Medicine and Epidemiology, University of Oslo, Oslo, Norway; c University of British Columbia, Division of Neurology, Vancouver, Canada; d The George Institute for Global Health, Royal Prince Alfred Hospital, Sydney, NSW, Australia; e Department of Medicine, Division of Clinical Pharmacology, Vanderbilt University School of Medicine, Nashville, TN.

**Keywords:** ACE I, ARB, atrial fibrillation, CVD risk, hypertension, RAAS blockers

## Abstract

Recent studies have demonstrated that atrial fibrillation significantly increases the risk of adverse clinical outcomes in high cardiovascular disease risk subjects. Application of renin–angiotensin–aldosterone system blockers for prevention of recurrence of atrial fibrillation and adverse clinical outcomes in subjects with atrial fibrillation is a theoretically appealing concept. However, results of clinical trials evaluating the effect of renin–angiotensin–aldosterone blockers on adverse clinical outcomes in high cardiovascular disease risk subjects with atrial fibrillation remain inconclusive.

A pooled study of 6 randomized controlled trials assessing the efficacy of renin–angiotensin–aldosterone blockers on subjects with atrial fibrillation was performed.

A total of 6 randomized controlled trials enrolled a total of 53,510 patients followed for 1 to 5 years. RAAS blockade therapy was associated with 14% reduction in the incidence of heart failure (OR: 0.86, [95%CI: 0.76– 0.97], *P*=0.018) and 17% reduction in the incidence of CVE (OR: 0.83, [95%CI: 0.70–0.99], *P* = 0.038). The corresponding decline in absolute risk against heart failure (ARR: 1.4%, [95%CI: 0.2–2.6%], *P* = 0.018) and CVE (ARR: 3.5%, [95%CI: 0.0–6.9%], *P* = 0.045) in the AF group was much higher than the non-AF group for heart failure (ARR: 0.4%, [95%CI: 0.0–0.7%], *P* = 0.057) and CVE (ARR: 1.6%, [95%CI: –0.1% to 3.3%], *P* = 0.071). No significant effect was noted on all-cause or cardiovascular mortality, stroke, or myocardial infarction.

This study suggests that RAAS blockade offers protection against heart failure and cardiovascular events in high cardiovascular disease risk subjects with atrial fibrillation.

## 1 Introduction

The application of renin–angiotensin–aldosterone system (RAAS) blockers in subjects with traditional cardiovascular disease (CVD) risk factors is an area of high interest, and recent studies have suggested therapeutic benefit of RAAS blockers in high CVD risk subjects.^[1,2]^ Recent studies in high CVD risk subjects without heart failure have suggested therapeutic benefit of RAAS blockade.^[3,4]^ Atrial fibrillation is the most common form of arrhythmia that is expected to rise further in prevalence.^[5]^ Recent studies have suggested that atrial fibrillation contributes substantially to the risk of adverse clinical outcomes such as heart failure, acute myocardial infarction, and major adverse cardiovascular outcomes.^[6–10]^ Two recent trials reported that RAAS blockers suppress atrial fibrillation more effectively than beta blockers and calcium blockers in hypertensive population.^[5,8]^ A previous meta-analysis also suggested beneficial effects of RAAS blockers in preventing the onset and recurrence of atrial fibrillation.^[4]^ Randomized controlled trials (RCTs) evaluating the effect of renin–angiotensin–aldosterone system blockers on clinical outcomes in subjects with atrial fibrillation were of limited power and yielded inconclusive results. Thus, whether the inhibition of atrial fibrillation translates into actual clinical benefit remains unknown. Further, whether blood pressure reduction results in comparable clinical improvement in subjects with and without atrial fibrillation remains unknown. Therefore, we aimed to evaluate the effect of RAAS blockers in high CVD risk subjects with atrial fibrillation (AF) and compared it with that of non-AF subjects from the respective trials.

## 2 Methods

### 2.1 Data sources and search strategy

A meta-analysis was performed according to PRISMA guidelines (Preferred Reporting Items for Systematic Reviews and Meta-Analyses).^[11,12]^ PubMed and EMBASE databases were searched using the keywords “angiotensin,” “angiotensin converting enzyme inhibitors ,” “angiotensin receptor blockers,” “individual names in these drug classes,” “hypertension,” “CVD risk,” “diabetes,” “atrial arrhythmia,” “atrial fibrillation,” and “randomized controlled trials.” Additionally, references of retrieved articles were manually searched to identify studies not captured by our primary search strategy. No restrictions were imposed on language and dates of publication. The final search was run on September 28, 2015. Ethical approval was not required as the study was based on published data and did not have direct access to patient information.

### 2.2 Study selection

**Figure 1:**
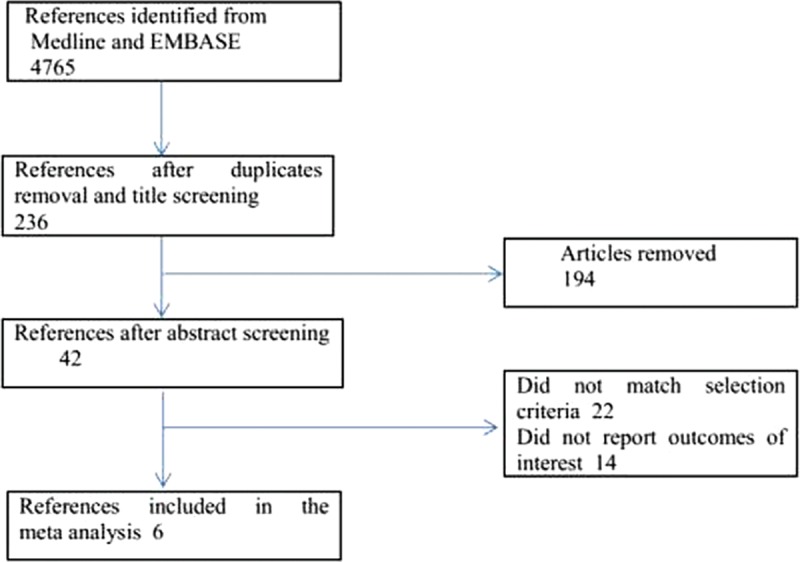
Flow diagram of study selection.

The flow diagram of study selection is shown in Fig. 1. Randomized controlled trials (RCTs) in subjects with hypertension and/or traditional CVD risk factors were screened for potential inclusion. Exclusion criteria are as follows: RCTs not reporting the outcomes of interest in AF cohort, lacking a comparator arm of non-RAAS blocker class or placebo, heart failure trials, trials with <100 participants and/or 10 events, case reports, reviews, and follow-up duration <1 year.

### 2.3 Endpoint definition

Endpoints of this study included “All cause mortality,” “Cardiovascular mortality,” “Heart failure,” “Stroke,” “Acute myocardial infarction (AMI),” and Cardiovascular events (CVE), a composite of stroke, heart failure, AMI, or cardiovascular death. Although the endpoint definitions varied slightly across the individual trials, they were the same for the 2 treatment arms and AF and non-AF groups.

### 2.4 Data extraction and quality assessment

Data were extracted by 2 independent reviewers (SC and LYS) and disagreements were resolved by consensus. Risk of bias among the included trials was assessed using standard criteria recommended by the Cochrane Collaboration.

### 2.5 Data synthesis and statistical analysis

The data were pooled across studies and analyzed using random effects meta-analysis models with inverse variance weighting. These are presented as ORs with 95% confidence intervals (CIs). The magnitude of statistical heterogeneity within AF and non-AF subgroups was estimated using the *I*^2^ statistic. *I*^2^ =  < 50%, 50% to 74%, and ≥75% was considered low, moderate, and marked heterogeneity, respectively. Tests of the homogeneity of treatment effects between AF and non-AF subgroups were performed by adding an interaction term to the statistical model. D-Stat, Software for the Meta-analytic Review of Research Literatures, was used to generate aggregate mean and standard deviation for age of the case and controls for each study. All other statistical analyses were performed using the STATA software 12.0. All *P*-values are 2-tailed with the statistical significance set at 0.05.

GISSI-AF did not report the data on heart failure, acute myocardial infarction, and cardiac mortality, whereas only the data for cardiovascular event was available in the AF group for the VALUE trial. Overall trial data for the cardiovascular event were substituted for the non-AF group for LIFE and VALUE trials.

## 3 Results

### 3.1 Study selection and characteristics

Of the initial 4765 studies identified by our primary search strategy, 42 articles were retrieved for detailed evaluation for potential inclusion of which 6 trials meeting the inclusion and exclusion criteria were included in the analysis (Fig. 1). Table 1 summarizes the basic characteristics of the included studies. In total, 6 randomized controlled trials^[5,8,9,11,13–18]^ enrolled a total of 53,510 patients, followed up for an average of 1 to 5 years. Preliminary small randomized controlled trials were followed by well-designed larger studies, and included patients with CVD risk factors such as hypertension, diabetes mellitus, diabetic nephropathy, left ventricular hypertrophy, left atrial enlargement, and coronary heart disease. PROGRESS and ADVANCE used ACE I and diuretic combination. Risk of bias among the included trials was generally low across the trials included.

**Figure 2:**
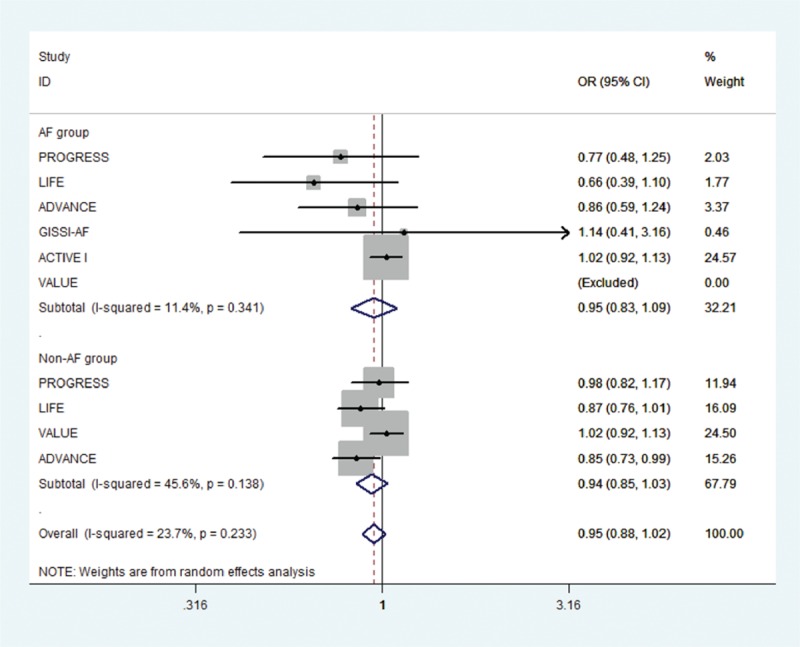
Effect of RAAS blockers on all-cause mortality.CI = confidence interval, OR = odds ratio, RAAS blockers = renin–angiotensin–aldosterone system blockers.

**Figure 3:**
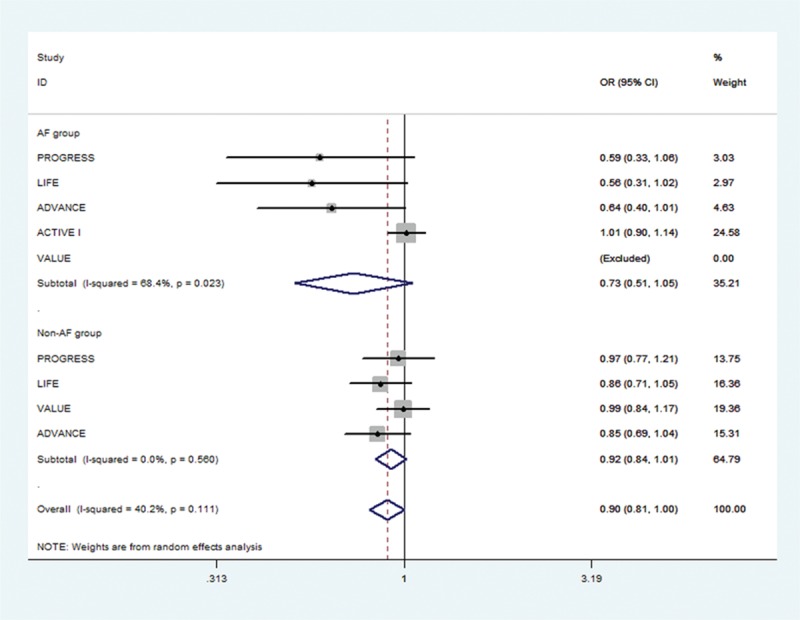
Effect of RAAS blockers on cardiovascular mortality. CI = confidence interval, OR = odds ratio, RAAS blockers = renin–angiotensin–aldosterone system blockers.

**Figure 4:**
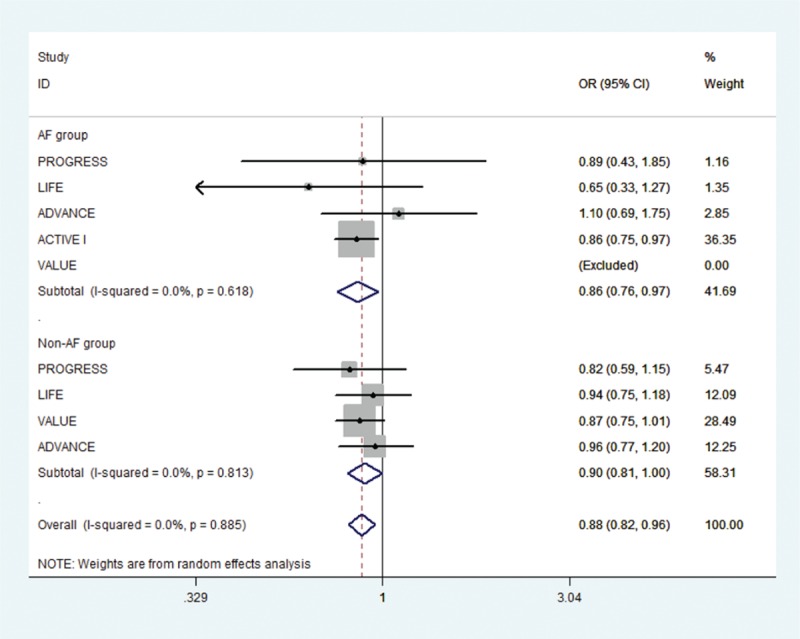
Effect of RAAS blockers on heart failure. CI = confidence interval, OR = odds ratio, RAAS blockers = renin–angiotensin–aldosterone system blockers.

**Figure 5:**
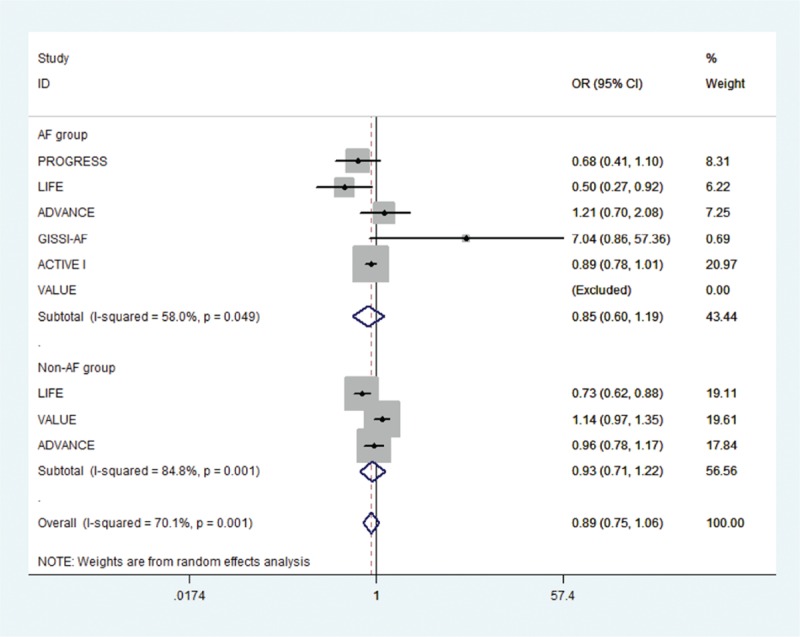
Effect of RAAS blockers on stroke. CI = confidence interval, OR = odds ratio, RAAS blockers = renin–angiotensin–aldosterone system blockers.

**Figure 6:**
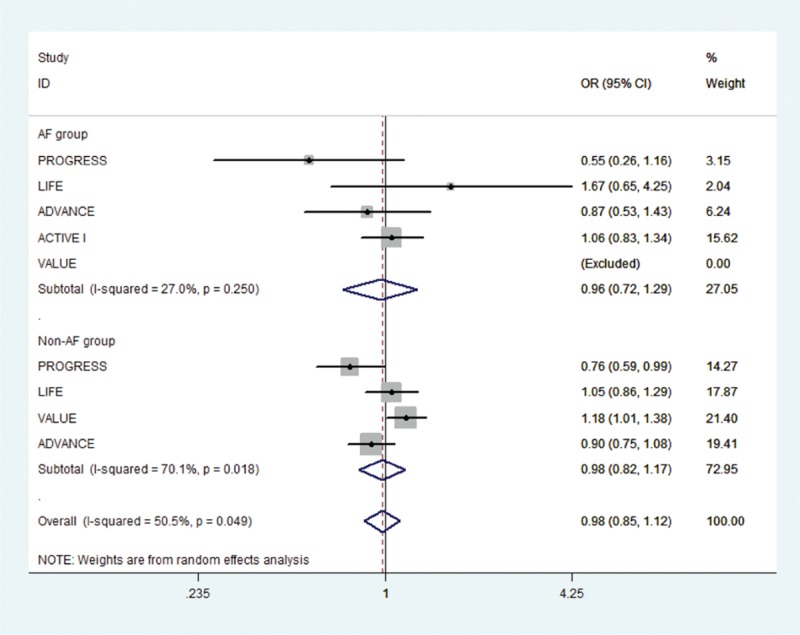
Effect of RAAS blockers on acute myocardial infarction. CI = confidence interval, OR = odds ratio, RAAS blockers = renin–angiotensin–aldosterone system blockers.

**Figure 7:**
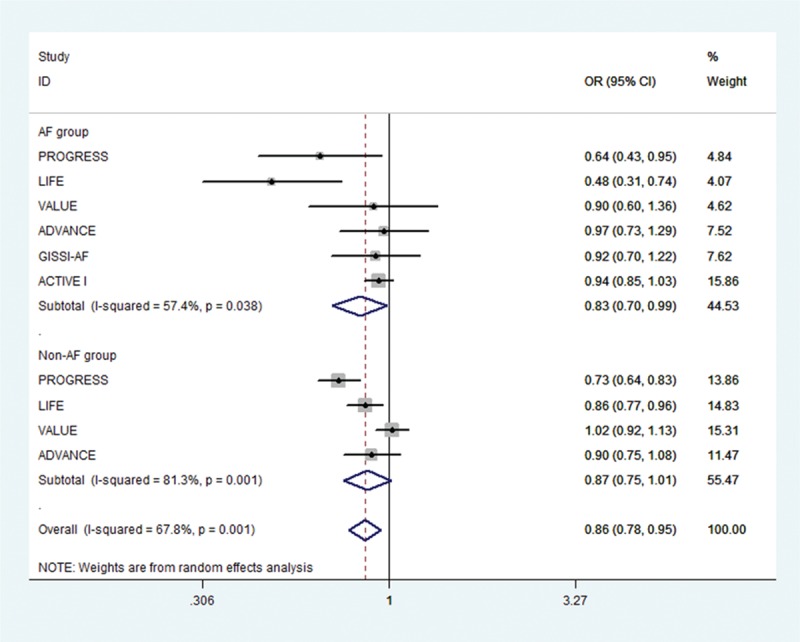
Effect of RAAS blockers on cardiovascular event. CI = confidence interval, OR = odds ratio, RAAS blockers = renin–angiotensin–aldosterone system blockers.

1. *All-cause mortality:* no significant effect of RAAS blocker therapy was seen on all-cause mortality in AF (OR: 0.95, [95%CI: 0.83–1.09], *P* = 0.468) or non-AF groups (OR: 0.94, [95%CI: 0.85–1.03], *P* = 0.174) (Fig. 2).
2. *Cardiovascular mortality:* no significant effect was seen in the AF group (OR: 0.73, [95%CI: 0.51–1.05], *P* = 0.090) or the non-AF group (OR: 0.92, [95%CI: 0.84–1.01], *P* = 0.095). The confidence interval was incredibly wide and trials were moderately heterogeneous in the AF group (*I*^2^: 68.4% and *P* = 0.023) (Fig. 3).
3. *Heart failure:* RAAS blocker therapy was associated with 14% reduction in the incidence of heart failure in AF (OR: 0.86, [95%CI: 0.76–0.97], *P* = 0.018) and a modest protective effect in the non-AF group (OR: 0.90, [95%CI: 0.81–1.00], *P* = 0.044) without any evidence of heterogeneity (Fig. 4). The test for interaction was borderline statistically significant (*P*_homogeneity_ = 0.090). RAAS blocker therapy was associated with a much greater decline in absolute risk against heart failure in the AF group (ARR: 1.4%, [95%CI: 0.2–2.6%], *P* = 0.021) than the non-AF group (ARR: 0.4%, [95%CI: 0.0–0.7%], *P* = 0.057).
4. *Stroke:* no significant treatment effect was observed in the AF group (OR: 0.85, [95%CI: 0.60–1.19], *P* = 0.340) or the non-AF group (OR: 0.93, [95%CI: 0.71–1.22], *P* = 0.597) (Fig. 5). The confidence interval for the AF group was very wide and trials were moderately heterogeneous (*I*^2^: 58% and *P* = 0.049), whereas non-AF trials were markedly heterogeneous (*I*^2^: 84.8% and *P* = 0.001).
5. *Acute myocardial infarction:* no significant effect of RAAS blocker therapy was seen in the AF group (OR: 0.96, [95%CI: 0.72–1.29], *P* = 0.800) or the non-AF group (OR: 0.98, [95%CI: 0.82–1.17], *P* = 0.794) (Fig. 6).
6. *Cardiovascular event (CVE):* RAAS blocker therapy was associated with 17% reduction in the incidence of CVE (OR: 0.83, [95%CI: 0.70–0.99], *P* = 0.038) in the AF group and a modest trend toward protection in the non-AF group (OR: 0.87, [95%CI: 0.75–1.01], *P* = 0.071) with marked heterogeneity in the AF group (*I*^2^: 81.3% and *P* = 0.001) (Fig. 7). The test for interaction was borderline statistically significant (*P*_homogeneity_ = 0.059). RAAS blocker therapy was associated with a greater decline in absolute risk against cardiovascular events in the AF group (ARR: 3.5%, [95%CI: 0.0–6.9%], *P* = 0.045) than the non-AF group (ARR: 1.6%, [95%CI: –0.1% to 3.3%], *P* = 0.071).
7. *Publication bias:* No evidence of publication bias was detected by Begg's or Egger's test for all-cause mortality (*P* = 0.153), heart failure (*P* = 0.725), stroke (*P* = 0.320), myocardial infarction (*P* = 0.591), and cardiovascular event (*P* = 0.233), but there was evidence of publication bias for cardiovascular mortality (*P* = 0.002).

**Table 1:**
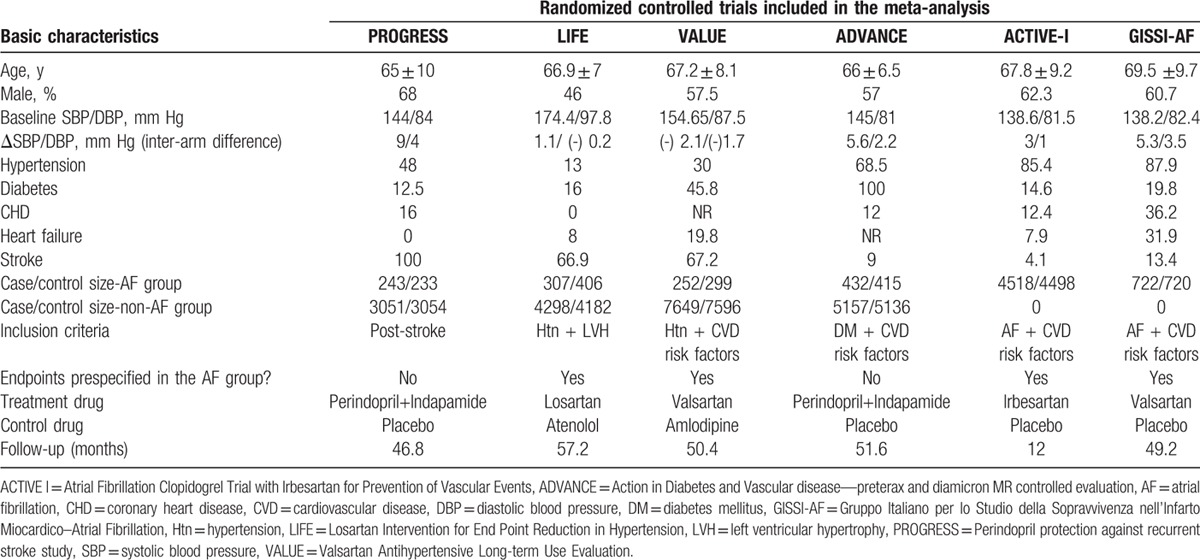
Basic characteristics of included studies.

## 4 Discussion

The current meta-analysis of 53,510 patients from 6 randomized controlled trials suggests that RAAS blockers offer protection against heart failure and cardiovascular event in high CVD risk subjects with atrial fibrillation. A total of 14% reduction in the incidence of heart failure (OR: 0.86, [95%CI: 0.76–0.97], *P* = 0.015) and 17% reduction in the incidence of CVE (OR: 0.83, [95%CI: 0.70–0.99], *P* = 0.038) was observed in the AF cohort, whereas the therapeutic benefit in the non-AF cohort failed to reach conventional limits of statistical significance for CVE with a modest 10% reduction in the risk of heart failure that was of borderline statistical significance. The results further suggested that subjects with atrial fibrillation derive comparable relative risk reduction but greater degree of absolute risk reduction against heart failure (ARR: 1.43%, [95%CI: 0.24–2.62%], *P* = 0.018) as well as CVE (ARR: 1.91%, [95%CI: 0.42–3.4%], *P* = 0.012) owing to their higher baseline risk. Modest blood pressure reduction and within trial interarm difference in blood pressure reduction with low event rates in the AF group resulted in wider confidence intervals. Although some of the trials were limited in size and some posthoc data were included in the current analysis, the relative effect of RAAS blockade in the AF group was comparable, in direction and magnitude, to non-AF cohort of the respective trials as well as the a priori designed ACTIVE I trial.

LIFE and VALUE trials provided evidence that RAAS blockers suppress the onset of atrial fibrillation more effectively than beta blockers and calcium blockers, respectively.^[8,15]^ Pooled estimate from the available reports on hypertensive and or high CVD risk subjects with atrial fibrillation suggests reduction in the risk of cardiovascular events, whereas the larger non-AF cohort of these trials showed a modest trend toward protection, highlighting the importance of RAAS blockade for blood pressure optimization in the AF cohort. A modest trend toward protection against CVE for AF cohort was noted in VALUE, likely due to the low number of events in the AF cohort and slightly higher blood pressure in Valsartan arm. In hypertensive patients with left ventricular hypertrophy (LVH), Losartan appeared superior to atenolol and this was especially evident in AF cohort. All the 4 placebo-controlled trials favored RAAS blockers with a larger effect size seen in the AF than non-AF group in the respective trials. ACTIVE I and GISSI-AF were placebo controlled secondary prevention trials that failed to show a statistically significant risk reduction in atrial fibrillation recurrence or cardiovascular events. It is noteworthy that majority of participants enrolled in these trials had advanced AF (persistent or permanent) and baseline use of background therapy with antiarrythmic drugs and multiple antihypertensive drugs including angiotensin-converting enzyme inhibitor (ACE I) was high with a modest difference in interarm BP during the trial. However, ACTIVE I still suggested a statistically significant 14% reduction in heart failure admission, and recurrent events analysis showed 11% reduction in the risk of composite end point of stroke, MI, or death from vascular causes. It also reported trends toward fewer stroke, transient ischemic attacks, and systemic embolization with the composite reaching a significance level, albeit in posthoc analysis. GISSI-AF did not report a difference in the hospitalization rate for cardiac or noncardiac events. However, it observed fewer events in a relatively smaller sample followed for a shorter duration (1 year), and the outcome on heart failure was not reported. Similar benefits of RAAS blockade were also seen in heart failure subjects with atrial fibrillation in the CHARM trial.^[19]^ One mechanism to account for superior efficacy of RAAS blockers is greater regression of cardiac remodeling including structural remodeling suggested by greater reduction of left ventricular hypertrophy and left atrial enlargement in LIFE, VALUE, NTP-AF^[20]^ and CASE-J,^[21]^ and electrical remodeling.^[22–26]^

### 4.1 Limitations

Drawbacks specific to the current meta-analysis include small sample size in the AF group, inclusion of some posthoc data and intertrial variations in inclusion/exclusion criteria. As data on cardiovascular events for non-AF group were not reported for LIFE and VALUE, overall trial data were used which may have affected the results slightly. However, considering the low event rate and the size of AF group in these trials, it is unlikely to change our conclusions. Significant intertrial variations in inclusion criteria poses a challenge to precise estimation of effect size in different subgroups such as hypertension, poststroke, and so on. but the similarities in the effect size between them enforces their generalization to AF subjects with CVD risk factors irrespective of the presence of hypertension. Results of PROGRESS suggested that the treatment effect is not affected by the presence of hypertension or application of anticoagulant therapy. Although ADVANCE and PROGRESS used Perindopril–Indapamide combination, the inter arm difference in BP reduction was small and comparable to monotherapy ARB trials. In an analysis using Perindopril monotherapy and single placebo arm data from PROGRESS, the results were slightly attenuated with 14% and 15% reduction in the risk of heart failure and CVE, respectively. The difference in effect size between Perindopril monotherapy and Perindopril–Indapamide combination therapy in PROGRESS only highlights the need of blood pressure optimization using combination antihypertensive therapy.^[10]^ Due to limited data, a comparison between ACE I and ARB could not be performed.^[27]^

## 5 Conclusion

Evidence from available studies suggests the application of RAAS blockers in high CVD risk subjects is associated with reduction in the risk of heart failure and cardiovascular events. The therapeutic benefit appears greater for subjects with atrial fibrillation compared to subjects without atrial fibrillation. Combination therapy with Indapamide appears to further reduce the risk by enhancing blood pressure control. With the failure of ARBs to suppress AF recurrence in a priori designed trials such as ACTIVE I and GISSI AF, future trials of RAAS blockers in AF subjects is unlikely. Thus, our results provide useful information to guide initial antihypertensive therapy in high CVD risk subjects with atrial fibrillation.
